# An Analysis of a Dengue Outbreak at a Large Hospital and Epidemiological Evidence for Nosocomial Dengue

**DOI:** 10.1155/2018/9579086

**Published:** 2018-06-26

**Authors:** N. D. B. Ehelepola, W. M. C. M. Wijesinghe

**Affiliations:** Teaching (General) Hospital–Kandy, Kandy, Sri Lanka

## Abstract

Reports on dengue outbreaks at hospitals are extremely rare. Here the authors analyze a dengue outbreak at the Teaching Hospital-Kandy (THK), Sri Lanka. Our hypothesis was that the present outbreak of dengue was due to nosocomial infections. Our objectives were to illustrate epidemiological evidence for nosocomial dengue infections among THK workers and comparison of dengue incidence of hospital workers of wards that treat dengue patients with workers of other wards, to ascertain whether most nosocomial dengue incidences occur closer to where dengue patients are treated and vector larvae were detected, and to draw the attention of the medical community to the significance of hospital outbreaks, making suggestions on how to improve dengue preventive work at the THK. We calculated weekly dengue incidences for the hospital workers and for the surrounding Kandy district population, plotted epicurves, and compared them. We also compared these with the temporal changes of numbers of patients who were admitted for other illnesses and then diagnosed with dengue and the numbers of containers with vector mosquito larvae found on hospital premises. Dengue incidence of the hospital workers for the 24-week study period (2388 per 100000 population) was significantly high when compared to incidence of the district (151 per 100000 population). Peaks of dengue incidence in hospital workers, the numbers of patients hospitalized for other illnesses contracting dengue, and numbers of containers with vector larvae occurred in the same week. The peak dengue incidence of the Kandy district happened six weeks later. There was no evidence to indicate blood contact causing dengue among hospital workers. The outbreak was controlled while dengue was rising in the district. This evidence indicates a probable nosocomial dengue outbreak. This outbreak adversely affected hospital workers, patients, and the community. We propose some measures to prevent such outbreaks.

## 1. Introduction

Dengue is a mosquito-borne emerging viral fever that can be life-threatening. The vector mosquitoes, genus* Aedes*, breed mainly in manmade water containers [1]. Approximately 96 million clinical dengue cases occur annually worldwide [1]. Despite that, only a handful of reports on dengue outbreaks at hospitals and nosocomial dengue are in medical literature [2–5]. There are past reports of other mosquito-borne nosocomial infections as well [6]. We used one definition of nosocomial infection accepted by the World Health Organization (WHO) for this study: an infection occurring in a patient in a hospital or another healthcare facility where the infection was not present or incubating at the time of admission. This includes infections acquired in the hospital but appearing after discharge and also occupational infections among staff of the facility [7]. There is a severe paucity of discussions of implications of hospital outbreaks of dengue in medical literature. Here we present an example of an outbreak of dengue in a large hospital (the Teaching Hospital-Kandy = THK) in a dengue hyperendemic country, Sri Lanka, in which the temporal sequence of events suggests occurrence of nosocomial dengue. Knowledge of the details of this outbreak may be useful to prevent and manage future outbreaks.

Using data from the Sri Lanka Department of Census and Statistics and the Ministry of Health, we estimated the notified dengue case incidence of the Kandy district for 2015 (the previous year) to give an idea of background to the reader. It was 93 per 100000 population. THK is usually the hospital that manages the largest number of dengue cases in the Central Province. Dengue incidence in Kandy is usually high during mid-November to mid-February and again in May-September [8, 9]. The onset of the rainy season, high mean temperature, high humidity, and the decline of diurnal temperature range several weeks before were demonstrated to facilitate this rise of dengue [8, 9] and this rise became augmented during the years when a new genotype of the virus was introduced to the population [8]. At present, there are no facilities locally to virologically prove nosocomial infection by molecular genetically demonstrating the same dengue virus in suspected nosocomial dengue cases, the salivary glands of vector mosquitoes caught on hospital premises and from some patients at THK.

Workers of the THK and other large Sri Lankan hospitals in cities are at a greater risk of contracting dengue than the general population based upon the following facts:* Aedes* vector indices are high in Kandy [10, 11] and in other populated areas of Sri Lanka [12]. Hospitals and other public places in Kandy and Sri Lanka have been revealed to be good breeding grounds for the* Aedes* vector by past studies [3, 10, 12]. Workers of THK and other large hospitals in Sri Lanka constantly live among patients with dengue viremia in cramped environments. All four serotypes of dengue virus cocirculate here. If hospital workers get asymptomatic seroconversions/a mild form of dengue infections first, they are more likely to get a severe disease when reinfected by another serotype [13].

## 2. Materials and Methods

### 2.1. Study Setting

The THK (N7.286318, E80.631651, and 516 m) premises cover 14.6 hectares. During the month of May 2016, 57107 inpatients were managed at THK, the outpatient department (OPD) has treated 32553 patients, and the clinics have treated 77258 patients. The total number of employees in same month was 5569 including student nurses. Dengue patients are usually treated in two pediatric and six internal medicine wards. In the THK air conditioning is limited mainly to intensive care units (ICUs) and operating theatres and mosquito screens are not in place. Therefore vectors can fly in to and out of wards. Most dengue patients treated here are residents of the Kandy district. A hospital dengue preventive program has been active since 2012, in addition to the national and municipal council programs.

### 2.2. Objective and Hypotheses

This is a retrospective, descriptive study. Our hypothesis was that the present outbreak of dengue among hospital workers was due to nosocomial (healthcare associated) infections. Our objectives were as follows:

- Main objective: illustrating epidemiological evidence for nosocomial infections among THK workers
- Comparison of weekly dengue incidence of THK workers with that of the Kandy district population
- Comparison of dengue incidence of hospital workers of wards that treat dengue patients with workers of wards without dengue patients. Expecting more workers to get dengue in the former
- To map the distribution of probable nosocomial dengue cases among hospital workers and patients hospitalized for other illnesses and then contracted dengue and sites where* Aedes* larvae were detected within the hospital and observe whether most nosocomial dengue occurs closer to where dengue patients are treated and larvae were detected
- To draw the attention of the medical community to the significance of hospital outbreaks of dengue by discussing how the present outbreak affected our hospital and the community
- Making suggestions on how to improve dengue preventive work at the THK.

### 2.3. Data

Dengue cases are diagnosed and managed at the THK based on the criteria of the 2012 national guidelines of the Health Ministry of Sri Lanka [14], which are broadly similar to the South-East Asia Regional Office of the WHO's 2011 comprehensive guidelines [13]. For this study, the case definition of a dengue case is a patient diagnosed of dengue and managed at the THK during our study period. All patients were not serologically (dengue NS1 antigen or IgM antibody for dengue) confirmed. However, all THK workers and patients diagnosed with dengue while being on treatment for another illness were serologically confirmed.

We gathered data from patients' notes and records at the infections control unit of the hospital from April 1st 2016 to September 16th 2016 period. The number of THK workers treated for dengue at THK, the buildings they work in most of the time, and the number of patients who contracted dengue while hospitalized for another illness (and the buildings where they were managed) were noted. The number (count) of hospital employees in May 2016 was obtained from the THK office and each relevant ward, and the estimated midyear population of the Kandy district for 2015 was obtained from the Sri Lanka Department of Census and Statistics. The reported number of dengue cases of the Kandy district during this period was obtained from the weekly epidemiology reports of the health ministry of Sri Lanka. The locations of the containers positive for* Aedes* mosquito larvae found on the hospital premises by the surveys conducted in the hospital during our study period were obtained from the Medical Officer of Public Health and the Public Health Inspector (PHI) of the THK. Those surveys covered the entire THK premises and were conducted with the help of the national Anti-Malaria Campaign (AMC) personnel according to their standard procedures. Identification of the species of larvae was confirmed by trained personnel of AMC. To get a basic idea of the impact of this, we gathered information on how this outbreak affected THK workers, patients, and the community by speaking to some THK workers, patients, and the Medical Officer of Health of Kandy city (the top public health official of the city).

### 2.4. Analysis

We estimated weekly dengue incidences for 100000, for the populations of hospital workers and the Kandy district population, created epicurves using Microsoft Excel 2007 software, and looked for evidence of nosocomial infections. We looked for supportive evidence for nosocomial dengue as well. Patients hospitalized for other illnesses developed dengue while at the hospital during the same period and presence of* Aedes* larvae at THK are good supportive evidence. Thus we studied temporal changes of numbers and locations of such patients and larvae for the period of the outbreak. We created a graph employing Microsoft Excel charts and created a spot map also for that. Then we estimated dengue incidence for the THK workers, THK workers who work at units treating dengue patients, and the rest of THK workers and for the population of the Kandy district and looked for statistically significant differences between them by calculating odds ratio and comparison of proportions between those groups. We employed Openepi 3.01 software for that.

## 3. Results and Discussion

### 3.1. Results

**Figure 1:**
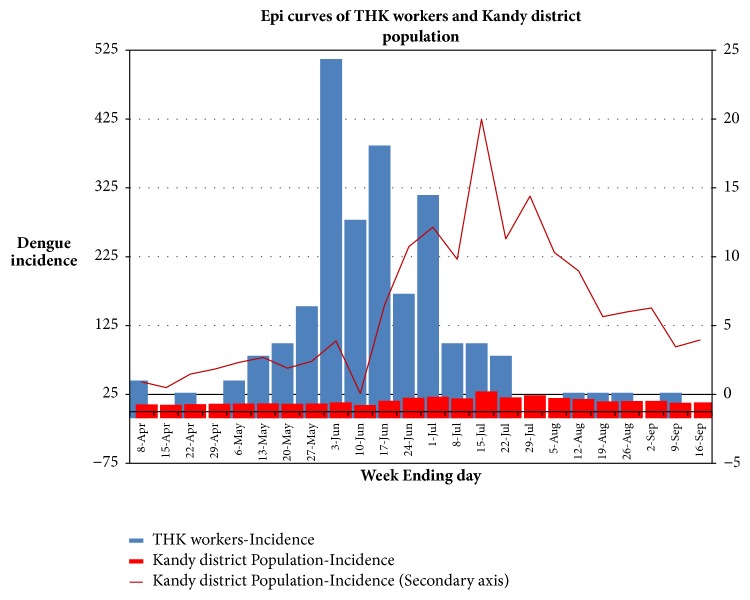
The epicurve. This shows the temporal sequence of weekly dengue incidences of the THK workers (blue bars) and the Kandy district population (red bars) and the weekly change in dengue incidence among the Kandy district population (red line). X-axis: the last day of the each week. Primary Y-axis: weekly dengue incidences (per 100000 population). Pattern of temporal changes of Kandy district population is not obvious here. Secondary Y-axis more clearly illustrates the pattern of temporal changes of the weekly dengue incidences of the Kandy district population in a different scale (red line).

Epicurves of Figure 1 show the temporal sequence of weekly dengue incidences of the THK workers and the Kandy district population and weekly change in dengue incidence among the Kandy district population.

The peak of the dengue incidence of THK workers occurred six weeks before the peak incidence of the Kandy district population. The differences in magnitudes of the dengue incidences of the two populations are also clearly seen in Figure 1.  Table 1 summarizes a comparison between THK worker and Kandy district populations.

133 THK workers and 2132 people of the Kandy district contracted dengue infections during the study period. The dengue incidences of THK worker population and the Kandy district population for the period of study (24 weeks) were 2388 and 151 per 100000 population respectively. When the comparison of proportions of dengue patients of the THK and the Kandy district was performed using Openepi 3.01 software, the P value was <0.001.

**Figure 2:**
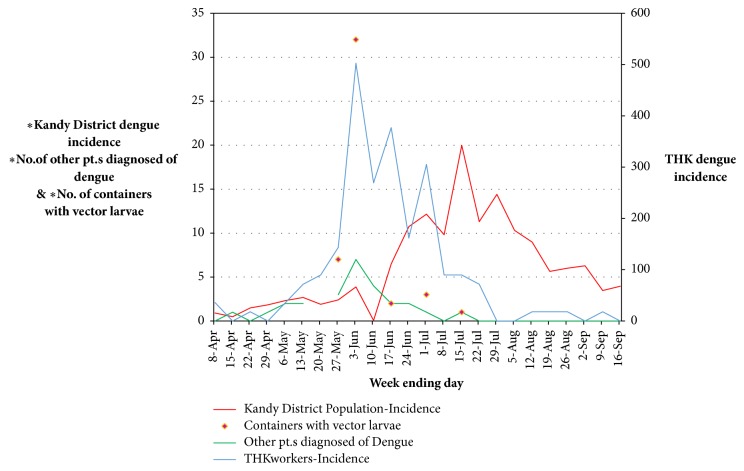
Temporal sequence of weekly dengue incidences of the THK workers, Kandy district population, counts of patients admitted to THK for other illnesses and then diagnosed with dengue each week, and the numbers of containers positive for vector mosquito larvae found in the hospital premises. X-axis: the last day of the each week. Primary Y-axis: dengue incidence per 100000 population in the Kandy district (in red), the numbers (counts) of patients admitted to THK for other illnesses and then diagnosed with dengue each week (in green), and the numbers of containers with vector larvae found in THK premises (in purple). Secondary Y-axis: dengue incidence per 100000 population in THK workers in a different scale (in blue).

The temporal sequence of dengue incidences of the THK workers, the Kandy district population, the counts of patients admitted to THK for other illnesses and then diagnosed of dengue, and the counts of containers positive for Aedes mosquito larvae found in the hospital premises are illustrated in Figure 2.

The graph of the count of patients admitted to THK for other diseases and then diagnosed of dengue each week shows the same temporal sequence pattern as the graph of weekly dengue incidences of the THK workers and both have peaks in the week ending June 3rd favoring the idea that both groups contracted dengue from the THK premises. Both graphs differ from the pattern of weekly dengue incidences of the Kandy district population. The peak of the dengue incidence of THK workers coincides with the week where the highest number of containers with* Aedes* larvae was found at THK premises during our study period.

**Figure 3:**
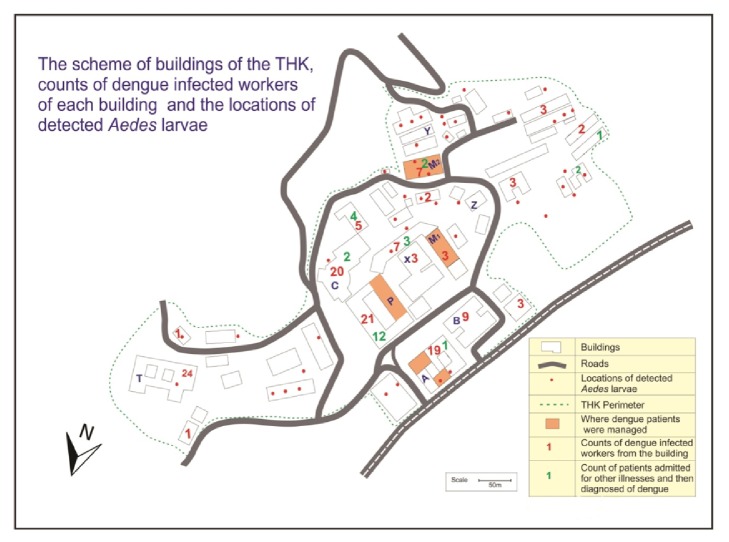
The scheme of buildings of the hospital (in mid-2016). In this scheme, the locations where dengue patients are managed are in tan color. A= building that houses the outpatients department. P= building that houses two pediatric wards. M1 and M2= buildings that house the internal medicine wards. T=nurses training school. The approximate locations of the water containers with Aedes larvae detected by entomological surveys are marked with red dots. Approximate scale of the scheme is given at the bottom.

Figure 3 is a scheme of buildings of the hospital that includes that house the internal medicine and pediatric wards, with dengue patients. Outpatients department (OPD) of the hospital also has high concentration of patients that come to the hospital with fever which includes dengue patients. The number of hospital workers infected with dengue working at each building is depicted in Figure 3 and the locations of containers with* Aedes* larvae were detected, also showing the duration of outbreak (from May 1st, 2016, to July 22nd, 2016).

Monthly total numbers of dengue patients warded at THK are depicted in Table 2.

The eight wards that manage dengue patients plus the OPD and emergency treatment unit have 504 workers. Out of that 17 had dengue. Out of 5065 other workers of the THK, 116 had dengue during our study period. The dengue incidence per 100000 population of workers of units treating dengue and rest of the THK workers were 3373 and 2291, respectively. Nevertheless there was no statistically significant difference in dengue incidence between the two categories (OR=1.49, 95% CI [0.89, 2.50]). Out of 2352 beds of the THK (that include few cots and incubators) 115 (<5%) were in the areas relatively protected from mosquito access such as ICUs and the premature baby unit. They were distributed among buildings A, B, C, P, X, and M_2_. During the period of April 1st–September 16th, 2016, 47% dengue cases of the THK were serologically (dengue NS1 antigen or IgM antibody for dengue) confirmed. The job categories of the 133 affected THK workers were as follows; 18 doctors, 39 nurses, 43 minor staff members (healthcare assistants), 19 student nurses and nursing tutors, and 14 workers of various other categories.

When asked about their concerns and views regarding this outbreak during first week of June 2016, 100 THK workers of all levels of hierarchy said that they were very worried about possibility of them contracting dengue. The first author in July 2016 asked all 48 staff members of one internal medicine ward how many times they contracted an illness that needed hospitalization as a probable result of working at the hospital (according to the best of their knowledge) during the last one year. No one had any such illness during the last year other than four members who were hospitalized with dengue in June. THK administration had to mobilize additional manpower from other wards to wards overcrowded with dengue patients where few workers are also on leave with dengue. The first author observed and some of these workers also agreed to taking some time to become familiarized with their new wards. In June 2016, the first author asked 33 patients who presented in late stages of several illnesses to an internal medicine ward for the reason for delay. One-third said fear of contracting dengue prevented them from coming earlier but aggravation of symptoms and inability to afford private treatment made them to come late. At the same month, some nondengue patients in internal medicine wards overcrowded with dengue patients went against medical advice telling fear of contracting dengue as the reason. In July 2016, 14 clinic patients on regular medical clinic follow-up who defaulted that clinic in June said fear of contracting dengue kept them away. The Medical Officer of Health of Kandy city personally communicated that she has noticed a rise of reported dengue cases from the immediate neighborhood of the hospital in May/June and believes that dengue has spread from the hospital.

## 4. Discussion

At the THK and most other hospitals of Kandy, dengue is diagnosed clinically as well by considering the patterns of the serial changes in platelet count, leucocytes count, hematocrit, and liver transaminases [13, 14]. Some cases are serologically confirmed. Recent studies indicate that after passing the threshold incidence of 150 per 100000 population during an epidemic (like in this outbreak) clinical diagnosis and serial blood counts are reliable ways to diagnose dengue [15]. Because dengue has been hyperendemic in Sri Lanka, our doctors are familiar with diagnosing dengue. Accordingly we think our data as fairly reliable. Nevertheless some other infections (Leptospirosis, Chikungunya) can give a similar clinical picture, hematological changes, and elevated liver enzymes to dengue. It is possible that a few cases of other infections were misdiagnosed as dengue because all dengue cases of the THK and the Kandy district were not serologically confirmed. However, all THK workers and patients diagnosed with dengue while being on treatment for another illness were serologically confirmed. Hence the data are very reliable.

As described above, different temporal patterns of dengue incidences in the epicurves (Figure 1) indicate a distinct outbreak of dengue among the hospital workers. There was a statistically significant difference in dengue incidence between the THK workers and the district population. This supports the idea that a nosocomial dengue outbreak has occurred among THK workers distinct from the district population. However, it should be reminded that THK workers were in the 18-60 age group and the district population consists of all age groups and many THK workers are included in the district statistics. The temporal sequence of numbers of patients admitted for other illnesses and then diagnosed of dengue (Figure 2) also supports the idea of nosocomial dengue outbreak. Blood contacts, mucocutaneous transmission, are also reported as a cause of nosocomial dengue in a clinical setting [4, 5]. None of the 133 hospital workers who contracted dengue have reported to the infections control unit any blood contacts with patients (needlestick/sharps injury) or undergone blood transfusions or surgery a month before their admission for dengue. Therefore, we conclude that they got infected via the* Aedes* vector.

The intrinsic incubation period (IIP) of dengue virus is usually considered 4-6 days [13]. The average mean temperature of the two closest weather stations to THK for a 10 year period was 25.1°C [9]. The extrinsic incubation period (EIP) of dengue is generally considered to be 4-10 days [1], but a review of studies done on the topic shows that EIP at 25°C can vary between 5 and 33 days [16]. The six-week delay between peaks of dengue incidence of the THK workers and district population is longer than the combined duration of IIP an EIP. The dengue incidence of THK workers started to rise from May 6th and reached the peak within four weeks. Interestingly, the number of containers with* Aedes* larvae also peaked in the same week of peak dengue incidence of THK workers indicating high vector density. We think the plausible explanation for the whole picture is an outbreak of nosocomial (healthcare associated) dengue.

During the first week of June THK dengue control program was intensified with outside help. Surveillance for mosquito breeding places conducted more frequently and vigorously in June and July. That also contributed to the identification of many containers positive for* Aedes* during that week. Figure 3 indicates that many THK workers who contracted dengue worked closer to wards with dengue patients and the OPD. Nonetheless, most THK workers do not have a fixed working spot. They move within their building usually and within THK premises sometimes while on duty. Most workers and visitors had entered and exited THK from gates several meters away from buildings A and P with dengue patients and vector breeding sites (Figure 3). Our results indicate that the odds of a THK worker of a unit treating dengue patients contracting dengue were higher compared to the rest of the hospital as we expected, but this observation was not statistically significant. As depicted in Figure 3 the vectors were abundant all over the hospital premises and that likely contributed to dispersing dengue. We would also like to state that the main administrative unit and the medical imaging unit of the THK are in building P (Figure 3). Hence, many hospital workers come there for administrative matters and patients for medical imaging and a few would have contracted dengue there.

Many workers of the nurses training school attached to the THK including 19 student nurses and tutors also contracted dengue (building T in Figure 3). That is one of the furthest places in the THK from the dengue patient concentrations. However,* Aedes* larvae were found there. Some of them appear to be infected when coming for ward work or from another student nurse or a worker with dengue viremia. Most patients diagnosed with dengue while being managed for another illness were also from the buildings near the building where dengue patients were managed (Figure 3). However, 12 out of 27 of such patients were diagnosed in fewer than six days after admission and hence probably got infected outside THK as well (usual IIP of dengue is 4-6 days). Despite the proximity to patients with dengue viremia and vector breeding sites, only 3 workers of building X (Figure 3) contracted dengue. That building houses many operating theatres, a few clinics, and the surgical ICU; hence most sections are air-conditioned. That may have restricted the access of mosquitoes to the building. Buildings Y and Z (Figure 3) are open wards in close proximity to patients with dengue viremia and vector breeding sites. However no dengue cases were reported from them. It is likely that some people in all buildings developed subclinical infections. According to statistics available at the THK records room, in 2016 the average duration of stay of inward patients was three days. Hence more patients may have contracted dengue while being treated for other diseases and gone home during IIP of dengue. Some patients (and some mothers who stayed with their sick children) went home and were then diagnosed of dengue within less than six days after but were not counted. That explains lesser number of patients diagnosed of dengue while being managed for another illness compared to the number of THK workers affected.

During first three months of 2016, when a comparatively smaller number of dengue patients were at hospital (Table 2) and when the local weather was unfavorable for dengue transmission [8, 9], no probably nosocomial dengue cases were reported. In May 2016, there were larger numbers of dengue patients at THK than the preceding four months combined, and in June even more dengue cases were there. Considering all, we believe that when vectors or patients with dengue viremia or especially when both become abundant at THK more nosocomial dengue cases ensue. The weather is favorable for dengue transmission and the intensity of the preventive work also influences nosocomial dengue incidence. As citizens living in Kandy for several decades and as mentioned in introduction we know that the dengue control work within THK premises is usually more intense than the surrounding area. However, concentration of patients with dengue viremia and susceptible hosts make it easier for vectors to transmit dengue at the THK and hence make the hospital more vulnerable for dengue outbreaks. This outbreak indicates the need for further improvements of THK dengue preventive work and the necessity of vigorous dengue control programs based upon integrated vector management principal (IMV) [13] at hospitals in dengue endemic areas especially during epidemics to prevent similar outbreaks.

The cornerstone of dengue prevention in THK is the elimination of breeding sites and immature forms of vectors. The hospital cleaning service removes all containers mosquitoes can breed in that they encounter on their daily sweeping of the premises but this collection is not recorded. There are random surveys covering THK premises specifically looking for mosquito breeding sites conducted by hospital PHI's team with the participation of AMC personnel and their findings are recorded. In addition, there are voluntary dengue circles in each ward and members randomly look for mosquito breeding sites in and around their wards. These surveys become more frequent once they detect unusually large numbers of mosquito breeding places and if several hospital workers or patients contract dengue. Dengue patients are provided with beds with mosquito nets to prevent transmission from them, but usually (especially during epidemics) the number of patients exceeds the number of beds in internal medicine and pediatric wards and many patients do not always utilize the nets provided. Once a probable nosocomial dengue case is identified, thermal fogging is employed to kill infected vectors in the vicinity. From the first week of June all these control measures were intensified with outside help; for example, fogging of the THK premises was performed twice a week for two months. Placement of ovitraps in selected locations to monitor the vector was introduced later. Considering results of some past studies we think that creation of additional barriers against dengue transmission inside the hospital by installation of screens against mosquitoes especially in units where dengue patients are managed and application of topical mosquito repellents to all potential dengue patients always and to the hospital workers during epidemics too may be useful. [9]. For example, first author has brought a bottle topical mosquito repellent (‘Soffell'-Diethyltoluamide 130g/l), kept it in the doctors' room of one internal medicine ward on May 15th, 2016, and invited 10 doctors of the ward to apply it morning and evening and no doctor developed clinical dengue in following 6 weeks. Out of 38 other staff members of the same ward four developed clinical dengue during this period and also one medical student and a student nurse. Three of them had onset of fever on the same day and the other three on the following day. In same week a container with larvae of both* Aedes aegypti* and* Aedes albopictus* was detected in this building. Those six people live in different areas and few weeks later they said public health teams have visited four of these persons' homes but could not find any mosquito breeding sites. These are strong evidence for nosocomial infections. Three out of those six obtained treatment from other hospitals closer to their homes thus they are not counted for this study. However, this is very basic evidence, not all doctors applied it regularly, and hence further studies are needed to prove efficacy of this method. Spraying of long acting insecticides to walls of hospital buildings and undersides of furniture and use of* Bacillus thuringiensis serotype H-14 *for vector larvae control in certain places may also be helpful [13].

Even though a much larger number of dengue patients were managed at the hospital in July and the weather was conducive for dengue transmission, infections among THK workers got controlled. The decline of dengue incidence of THK workers while that of the surrounding Kandy population rose further supports the idea that nosocomial dengue occurred and indicates that even during the height of an epidemic in the community, vigorous preventive methods can control nosocomial dengue at hospitals, and that is an important lesson to remember. Further strengthening of THK dengue preventive program with wider participation of all stake holders based upon IMV principals may be useful in prevention of further outbreaks. Dearth of funds for preventive work is a key issue in implementing additional preventive measures in Sri Lanka and in most other countries severely affected by dengue although prevention is better than treatment. According to one study, hospital management of an adult with dengue fever and severe dengue hemorrhagic fever, respectively, cost the health ministry of Sri Lanka about 196 and 887 US dollars but the expenditure on dengue prevention per year per reported case of dengue was only 97 US dollars in 2012 [17]. Hospital premises are small areas with large populations. Thus preventive measures can be implemented with less cost per head.

In 2009, 37 hospital workers and 21 patients being treated for other diseases acquired confirmed dengue from the National Hospital of Sri Lanka-Colombo [3]. However, that report does not elaborate on evidence for nosocomial infections. During the 2015 epidemic, four nosocomial dengue cases among patients admitted for other diseases were reported from Hospital das Clinicas, Sao Paulo, Brazil [2]. According to our literature survey they were the only two past reports about dengue acquired at hospitals via the* Aedes* vector. One Indian team in 2008 responding to an article in the Lancet journal reported of 21 serologically confirmed cases of nosocomial dengue in healthcare workers and suspected possible aerosol transmission [18]. However, the author of the original article disagreed and replied transmission by mosquitoes was more likely [18]. A Bangladeshi team publishing on hospital acquired infections in 2016 has warned of a big risk of nosocomial dengue in that country [19]. Both printed and electronic news media frequently report about possible hospital outbreaks of dengue indicating they are common. Here we cite three recent examples from Sri Lanka, from three (English) national newspapers [20–22]. Many healthcare workers we know and abroad believe highlighting nosocomial infections is pointless and that would tarnish the image of their workplace and create trouble for them and their coworkers [23]. We believe the utility of our analysis of this outbreak could outweigh any negative aspects of reporting it.

The magnitude of this outbreak motivated us to give a basic idea of its impact. The information we gathered by talking to those who were affected is very basic. However it may help others to foresee what to expect in future outbreaks in other hospitals. The media highlighted this outbreak and sensationalized a death of one hospital worker as negligence. That led to anxiety among THK workers and to the practice of defensive medicine by some of them. Further studies may help identify dengue as an occupational hazard of workers in large hospitals in endemic countries. During the height of this outbreak, the THK administration had to mobilize additional manpower to wards treating dengue patients. Some Kandy residents had a transient fear of utilizing the hospital. According to the Medical Officer of Health of the Kandy city dengue has spilled to the immediate neighborhood of the hospital. Transportation methods and hubs are known contributors of the global spread of dengue [24]. The premises of the main railway station of the Kandy city are across the road of the THK and the main bus terminal is adjacent to the railway station.

Vector breeding sites were all over the THK premises. Dengue virus can get vertically transmitted in the vector [6, 13]. Patients infected with different sero/genotypes of the dengue virus are present in THK like other major hospitals of dengue hyperendemic nations. Therefore, hospital workers are more prone to get coinfection with multiple sero/genotypes. That can lead to a recombination of genetic components and the emergence of more virulent viruses [25].

## 5. Limitations of the Study

Hospitalized dengue cases are only a fraction of total dengue cases. THK workers who were treated for dengue at other hospitals were not counted. Some patients who appeared to be infected while being treated at THK for other illnesses (and some bystanders who stayed with their sick children), went home, and were then diagnosed with dengue within less than six days were not counted. Some hospital workers and patients who developed fever at the hospital may have got infected outside the hospital. The diagnosis of dengue in all cases of THK and Kandy district was not serologically confirmed. Other factors such as window space available for vectors to fly in and out of each building and practices like using mosquito repellents may also have influenced the number of cases in different buildings but these factors were not assessed. Investigations of each and every hospital staff member and all dengue patients for a longer period may give a better picture of the situation but that task is beyond the capability of the authors.

## 6. Conclusions

The mid-2016 dengue outbreak at the THK affected hospital workers and users severely and is probably a nosocomial dengue outbreak. Such major outbreaks are likely to adversely affect the hospital workers, the smooth functioning of the hospital, and the communities they serve especially at a height of dengue epidemic, as was the case at the THK. Nonetheless such outbreaks can be controlled with additional effort. We have to generate more information on this issue from similar hospitals to confirm the findings of the present study and take steps to prevent dengue outbreaks at hospitals.

## Figures and Tables

**Table 1:** The comparison of THK worker and Kandy district populations.

	**THK Worker Population**			**Kandy District Population (2015)**
	Workers of units that manage dengue pt.s	Workers of other units	Total workers of the THK	

**Total population**	504	5065	5569	1416000

**Number of dengue cases**	17	116	133	2132

**No. of dengue cases as a percentage of the population**	3.37%	2.29%	2.34%	0.15%

**Dengue Incidence for our study period per 100,000 population**	3373	2291	2388	151

**Table 2:** Monthly totals of dengue patients warded at THK.

Month (of 2016)	January	February	March	April	May	June	July	August
Total of dengue patients warded at THK	42	28	38	42	159	555	658	296

## Supplementary Materials

Supplementary Materials Graphic abstract: this figure illustrates core contents of the paper. That is evidence for nosocomial dengue infections, adverse effects of outbreak, and control of the outbreak.

## References

[1] WHO (2017). Dengue and severe dengue. *World Health Organization*.

[2] Almeida-Nunes J., Marcilio I., Oliveira M. S. (2016). Hospital-acquired vector-transmitted dengue fever: an overlooked problem?. *Infection Control & Hospital Epidemiology*.

[3] Senaratne S. (2013). Dengue outbreak and management of control measures in the national hospital of Sri Lanka. *Sri Lankan Journal of Medical Administration*.

[4] Chen L. H., Wilson M. E. (2005). Nosocomial dengue by mucocutaneous transmission. *Emerging Infectious Diseases*.

[5] Nemes Z., Kiss G., Madarassi E. P. (2004). Nosocomial transmission of dengue. *Emerging Infectious Diseases*.

[6] González L., Ochoa J., Franco L. (2005). Nosocomial Plasmodium falciparum infections confirmed by molecular typing in Medellín, Colombia. *Malaria Journal*.

[7] Ducel G., Fabry J., Nicolle L. (2002). *Prevention of Hospital-Acquired Infections: a Practical Guide*.

[8] Ehelepola N. D. B., Ariyaratne K., Buddhadasa W. M. N. P., Ratnayake S., Wickramasinghe M. (2015). A study of the correlation between dengue and weather in Kandy City, Sri Lanka (2003 -2012) and lessons learned. *Infectious Diseases of Poverty*.

[9] Ehelepola N. D. B., Ariyaratne K. (2015). The interrelationship between dengue incidence and diurnal ranges of temperature and humidity in a Sri Lankan city and its potential applications. *Global Health Action*.

[10] Kusumawathie P. H. (2005). Larval infestation of aedes aegypti and Ae. albopictus in six types of institutions in a dengue transmission area in kandy, Sri Lanka. *Dengue Bulletin*.

[11] Weeraratne T. C., Perera M. D. B., Mansoor M. A. C. M., Karunaratne S. H. P. P. (2013). Prevalence and breeding habitats of the dengue vectors Aedes aegypti and Aedes albopictus (Diptera: Culicidae) in the semi-urban areas of two different climatic zones in Sri Lanka. *International Journal of Tropical Insect Science*.

[12] Louis V. R., Quiñonez C. A. M., Kusumawathie P. (2016). Characteristics of and factors associated with dengue vector breeding sites in the city of Colombo, Sri Lanka. *Pathogens and Global Health*.

[13] (2011). *Comprehensive Guidelines for Prevention and Control of Dengue and Dengue Hemorrhagic Fever*.

[14] (2012). *National Guidelines, Guidelines on Management of Dengue Fever & Dengue Haemorrhagic Fever In Adults*.

[15] dos Santos Carmo A. M., Suzuki R. B., Riquena M. M., Eterovic A., Sperança M. A. (2016). Maintenance of demographic and hematological profiles in a long-lasting dengue fever outbreak: implications for management. *Infectious Diseases of Poverty*.

[16] Chan M., Johansson M. A. (2012). The incubation periods of dengue viruses. *PLoS ONE*.

[17] Thalagala N. (2014). *Health System Cost for Dengue Control And Management in Colombo District, Sri Lanka 2012*.

[18] Gupta V., Bhoi S., Goel A., Admane S. (2008). Nosocomial dengue in health-care workers. *The Lancet*.

[19] Shahida S. M., Islam A., Dey B. R., Islam F., Venkatesh K., Goodman A. (2016). Hospital acquired infections in low and middle income countries: root cause analysis and the development of infection control practices in Bangladesh. *Open Journal of Obstetrics and Gynecology*.

[20] Warakapitiya K. (2016). Kalubowila Hospital facing crisis with increasing number of dengue patients. *SundayTimes-SriLanka*.

[21] Aloysius C. (2017). Crowded wards, staff shortages, inadequate ICUs in most hospitals: constraints overwhelm docs battling infectious diseases surge. *Sunday Observer-Sri Lanka*.

[22] Kurunegala Correspondent, More than 20 staff of K'negala hospital down with dengue, The Island. http://www.island.lk/index.php?page_cat=article-details&amp;page=article-details&amp;code_title=163890.

[23] Maciel A., Carvalho B., Padoveze M. (2015). Barriers to investigate and to report nosocomial outbreaks to health authorities in São Paulo, Brazil: a mixed method approach. *Antimicrobial Resistance and Infection Control*.

[24] Gubler D. J. (2011). Dengue, urbanization and globalization: the unholy trinity of the 21st century. *Tropical Medicine and Health*.

[25] Senaratne T., Sirisena P., Muruganathan K., Noordeen F., Carr J. (2016). Co-infections with multiple dengue virus serotypes in patients from 3 different Provinces of Sri Lanka, a dengue hyper endemic country. *International Journal of Infectious Diseases*.

