# Supplementary figures and images for: An Analysis of a Dengue Outbreak at a Large Hospital and Epidemiological Evidence for Nosocomial Dengue
Source: J Trop Med. 2018 Jun 26;2018:9579086. doi: 10.1155/2018/9579086 (PMC6038582; doi:10.1155/2018/9579086)

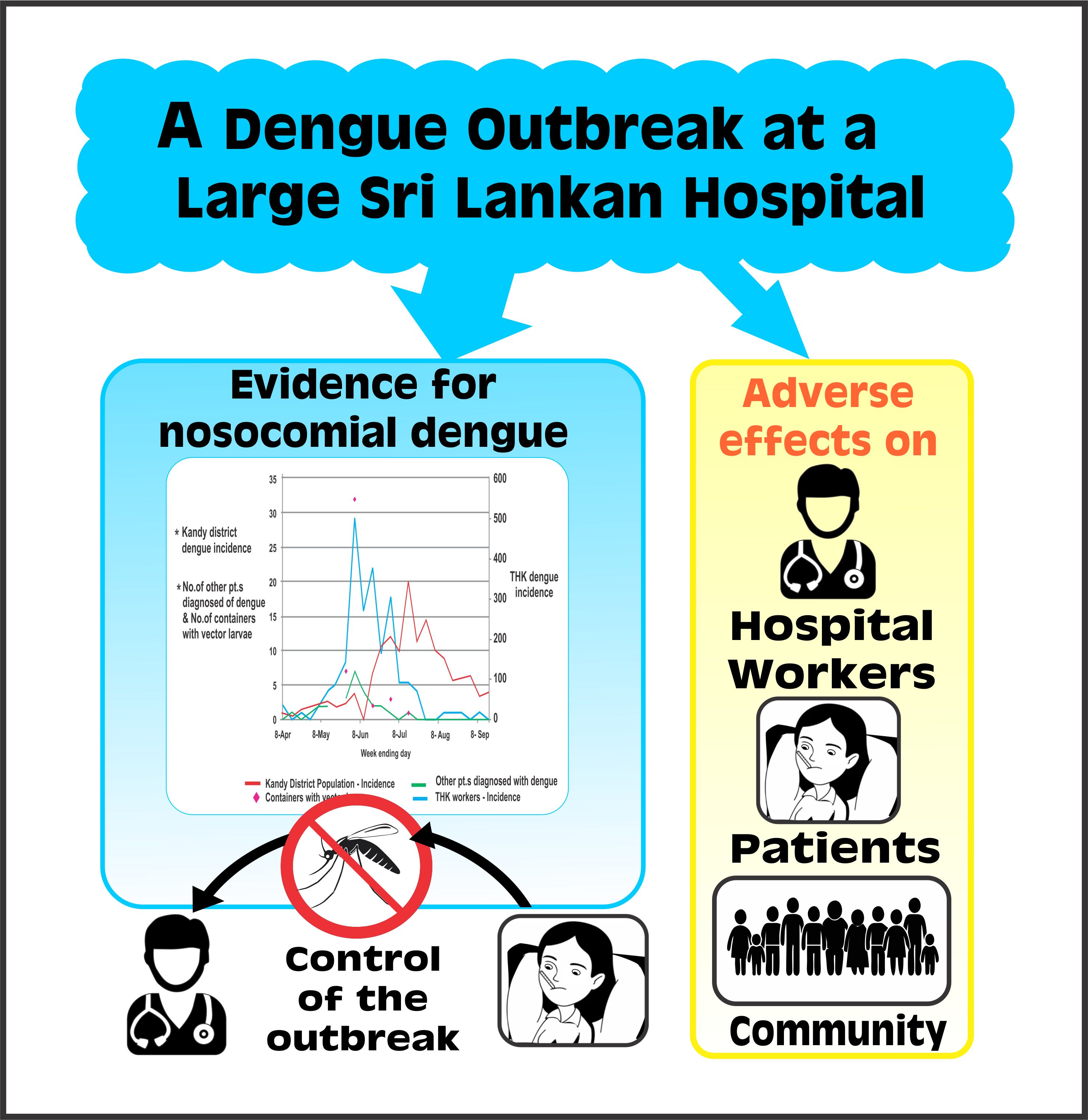

Supplement: Supplementary Materials — Graphic abstract: this figure illustrates core contents of the paper. That is evidence for nosocomial dengue infections, adverse effects of outbreak, and control of the outbreak. [file 9579086.f1.jpg]
